# Dr. Simeon Tucker Clark

**Published:** 1892-01

**Authors:** 


### Obituary.[1]

Dr. Simeon Tucker Clark, of Lockport, died in that city Thursday evening, December 24, 1891, of paralysis. Dr. Clark was attending physician to the Niagara County Almshouse, and while paying one of his accustomed visits to that institution, a day or two before his death, was stricken with the malady which finally proved fatal.

He was a member of the several County and State Medical Societies, and Professor of Medical Jurisprudence in the Medical Department of Niagara University. He had attained to eminence in his profession, and his poetical talents were of a high order. He acquired fame as an alienist, and his services were often sought in the courts as a medical expert in mental and criminal cases.

He leaves a widow, a son, and a daughter, to mourn his untimely death, and a profession that will everywhere unite in sympathy with those who have suffered this bereavement.

1. These notices were received too late to appear in their proper place.—[*Printer.*]

